# Quality assessment of pathologic data in cancer registry centers based on ICD-O-3

**DOI:** 10.22088/cjim.13.3.589

**Published:** 2022

**Authors:** Raziehsadat Mousavi, Ghahraman Mahmoudi, Hossein-Ali Nikbakht, Mohammad Ali Jahani

**Affiliations:** 1 Hospital Administration Research Center, Sari Branch, Islamic Azad University, Sari, Iran; 2 Social Determinants of Health Research Center, Health Research Institute, Babol University of Medical Sciences, Babol, Iran

**Keywords:** Neoplasms, Grade, Assessment, Accuracy, Reliability

## Abstract

**Background:**

Prerequisite for achieving the goals of the registration program is the existence of valid and accurate data, and the usability of this data is possible if they are coded correctly. This study assets the quality of pathological data of the population-based cancer registration centers based on ICD-O-3.

**Methods:**

This applied study was performed descriptively and retrospectively. The study population included 20129 pathology reports sent to the population-based cancer registration center of Mazandaran Province during 2018-2020. A total of 2015 out of, 2050 samples of the received reports were examined according to stratified random sampling method. A researcher checklist was made to collect the data, and STATA 13 and Cohen's Kappa agreement coefficient were used to analyze the data.

**Results:**

Among the 2015 reports of pathology, 1114 (55.3%) pathology reports were related to government centers, (42.9%) 865 cases were registered with their topographic code, morphology and behavior. Based on the registration of the exact topographic code, the kappa coefficient and the total agreement were 0.266 and 27.70%, respectively. Kappa coefficient in all received reports and reports with topographic code was 0.346 and 0.906, respectively. In the reports with topographic code, the most reports of cancers were related to cancers of the gastrointestinal organs (97.6%) 246.

**Conclusion:**

The accuracy of the codes given in the pathology centers in terms of topographic, morphological, behavioral and grade codes based on the percentage of agreement with the coding was above average, which were higher in governmental centers and also in some cancers.

Cancer is one of the most common non-communicable diseases and one of the most common diseases that in recent decades has been shocking statistics of mortality (1, 2). In Iran, cancer is one of the most common non-communicable diseases and after heart disease and road accidents is considered as the third leading cause of death (1), which makes it increasingly important for prevention, timely and correct diagnosis and finally treatment of this disease. Prevention and reduction of cancer cases requires the existence of cancer control programs, that the cancer registration system is one of the essential components of cancer control programs (2). The use of databases for information retrieval, epidemiological studies and management decisions is subject to correct clinical coding. Therefore, the accuracy of the coding of medical records is essential. As a result, the quality of the coded information must be evaluated, and these are the steps taken to ensure the quality of the coding. 

Existence of coding error leads to incorrect classification of diseases and consequently incorrect statistics. Relying on data whose classification quality is poor will pose risks to both care providers and managers in planning and policymaking, education and research, care delivery, and so on. By identifying the factors related to the accuracy of disease coding, the necessary information can be provided to perform useful and effective interventions to improve the quality of disease information (3, 4).

 The Cancer Registry Program is an information management system that collects, stores, manages, analyzes, and reports data on cancer patients and deaths from the disease (5, 6). On the other hand, the use of this information is possible when this information is properly organized, classified and coded (7, 8). 

In recent years, the process of coding medical records due to communication closeness to care quality issues and reported data has become increasingly important nationally and internationally (8, 9). The coding accuracy of medical records is essential, and the quality of the coded information must be evaluated to ensure quality (3, 10). To integrate the tumor coding process into cancer registration programs, the ICD-O International Coding System has been designed by the International Agency for Research on Cancer. In the National Cancer Registration Program of Iran, the ICD-O-3 coding system is used to classify and encode tumor information (11).

 Most of the studies performed on the evaluation of coding quality and accuracy show that the accuracy of coding of diagnoses is at a low level. In a study conducted in Germany, the reliability of coding of diagnoses was evaluated at a low to moderate level (12). 

The results of the study of Caski showed that the USA health care system used diagnostic codes for billing and reimbursement in its research and showed that all cancer records in these countries are high standard in terms of completeness and accuracy (13). 

The results of the study of Feiz showed that the average of accuracy of diagnostic accuracy was 80.3% and the average of accuracy of treatment measures was 84.2%. The reported accuracy also indicates that the data collected are typically strong enough to be used for research and managerial decision-making (14).

Therefore, considering the importance of coding information on cancer patients and the need for accuracy and precision of these codes, and considering that so far few studies have been done to evaluate and check the accuracy of information and codes of cancer patients' records and the status of information registration and information accuracy and the codes assigned to the pathology reports sent to the population-based cancer registration centers have not been reviewed, this study is done to determine the status of data registration of pathology reports sent to the population-based cancer registry center of Mazandaran province and qualitative evaluation of pathological data based on the international classification system of cancer diseases to identify probable errors and provide solutions to eliminate them to improve the quality of the registration data.

## Methods

This applied study was performed descriptively and retrospectively, after obtaining the code of ethics at IR.IAU.CHALUS.REC.1399.025 from Islamic Azad University and receiving a letter of introduction. The study population included, 20129 pathology reports sent by 32 government centers and 73 private centers active in 14 cities of the province, which were sent to the Cancer Registration Center during the years 2018-2020. Using Cochran's formula and also considering the confidence level of 0.95, study power of 0.90 and acceptable error of 0.03 for the ratio, 1900 samples were needed to check the error between the coders of the pathology centers and the coder of the cancer registration center. 

Therefore, about 10% of the samples of each year (three years studied) were considered as the final sample using stratified random sampling method. In such a way that by using proportional allocation and in proportion to the weight of the type of pathology center (government or private) in the research community, the sample size was determined for each year. Finally, 2050 pathology reports were reviewed in this study. 12 reports of them were received from out-of-university pathology centers to cancer registration centers and 23 reports were benign cancers, and they were excluded from the study, thus 2015 reports were considered in the final analysis. 

A researcher-made checklist was used to collect data, experts and thinkers were used to assess the validity of the checklist, and after corrections, they were approved. By assigning a record code to each report, all patient information was published without mentioning the names and identities of patients and was collected while maintaining the principles of security and confidentiality.

Next, by examining the site of the cancer as well as the histology and behavior of the cancer cell announced in the pathology report and selecting the site and the type of cancer, referring to the cancer coding book and according to the existing rules, the necessary codes for the organ with the tumor (topography) including cancer code and grouping, tumor histology type (morphology), tumor behavior (benign, in situ or malignant) and cancer tumor grade (grade) were extracted from the book and recorded in the relevant checklist. The accuracy of the codes was assessed using the Cohen's Kappa agreement coefficient. In general, this index shows the degree of agreement of the two evaluators (excluding chance) on a two-state trait. 

Kappa coefficient and statistical analysis based on it are numerical values ​​between -1 to +1, which the closer to +1 indicates the existence of a proportional and direct agreement. Values ​​close to -1 indicate inverse agreement, and values ​​close to 0 indicate disagreement. Kappa coefficient, measures reliability of agreement of different persons regarding the coding of a particular case (Inter-rater reliability) or the external reliability and agreement of a person at different times of coding in a particular case (Intra-rater reliability) or measures internal reliability. 

If the kappa coefficient is below zero, the degree of reliability is poor, between (0.00-0.20) low, (0.21-0.40) relatively poor, (0.41-0.60) moderate, (0.61-0.80) is acceptable and (0.81- 1.00) is almost complete. Kappa = Pi = (PA0 – PAE) / (1 – PAE)

A value of PA0 indicates the degree of agreement between the two assessors, and a value of PAE indicates the degree of agreement expected (15). In addition to calculating the kappa coefficient, the percentage of agreement was also reported. All analyses were performed using STATA software Version 13.

## Results

In this study, 2015 pathologic reports were examined, (0.57%) 1150 reports did not have topographic, morphology and behavior codes. In other words, 865 (42.9%) reports had topographic code, morphology and behavior codes. Also, only 659 (32.7%) reports had a grade code. 

The most reported reports in this study were from the cities of Sari, Amol and Babol and the numbers of these cities' reports were 648 (32.2%), 326 (16.2%) and 250(12.4%). Governmental centers had 1114(55.3%) reports, of which 496 (57.3%) reports, and they had the codes of topography, morphology and behavior. Based on the allocation of the accurate topographic codes in the 2015 report, the kappa coefficient and the total agreement were 0.266 and 27.70%, respectively, which indicates a lower-than-average accuracy. 

Among the cities, Babol had 0.690 (acceptable accuracy), and had the highest kappa coefficient. Of the 865 reported codes, the kappa coefficient and the total agreement, respectively; 0.632 (acceptable) and 64.51% and Tonekabon city with kappa coefficient of 0.837 (almost complete accuracy) is the highest and also Noor, Babol and Amol with kappa coefficients of 0.692, 0.690 and 0.679, were also in the next ranks in terms of accuracy. 

To investigate this case, instead of recording the exact code, only the registration of the cancer group was considered. The results showed that among the 2015 reports, the total kappa coefficient was 0.347, which indicates that the accuracy is lower than average. Among the cities, Babol had the highest kappa coefficient with 0.942 (almost complete) (Table 1). According to the standardized classification of cancers, the kappa coefficient in all received reports and reports with topographic codes was 0.346 (relatively good) and 0.906 (almost complete). In the reports with topographic codes, the most reported cancers (percentage of agreement) were related to gastrointestinal cancers 246 (97.6), breast cancers 84 (88.4), female genital cancers 72 (94.7) and skin cancers (table 2).

The results showed that the morphology code accuracy on all received reports and coded reports in pathology centers based on kappa coefficient were 0.331 and 0.834, showed a relatively good and almost complete accuracy, but the accuracy of the behavior and grade codes very low, even on the reported codes (table 3).

The results of the accuracy of the topographic, morphology, behavior and grade codes by center type (public and private), showed that the accuracy is less than average and in all reports, government reporting centers have a higher accuracy in registration. In the reported code, governmental centers almost performed better in registration. 

The accuracy of topographic code registration based on cancer grouping and morphology codes is almost complete, in grade code and in behavior code, the accuracy of registration is low (table 4).

**Table 1:** Frequency of reports and accuracy of topographic codes of pathology reports by city

**City**	**Total number of reports**	**Topographic, Morphology and Behavior codes**	**Based on accurate topographic code registration**			**Based on cancer grouping registration**		
	**Frequency (percentage)**	**Frequency( %)**	**Kappa coefficient** **(n=2015)**	**Kappa coefficient** **code declaration items (n=865)**	**Percentage of code declaration agreement** **(n=865)**	**Kappa coefficient** **(n=2015)**	**Kappa coefficient** **code declaration items (n=865)**	**Percentage of code declaration agreement** **(n=865)**
Ramsar	66(3.3)	65(7.5)	0.401	0.407	44.62	0.893	0.913	93.85
Tonekabon	214(10.6)	131(15.1)	0.501	0.837	84.73	0.558	0.955	96.18
Chalous	109(5.4)	23(2.7)	0.127	0.613	65.22	0.156	0.936	95.65
Nowshahar	35(1.7)	14(1.6)	0.160	0.398	42.86	0.281	0.878	92.86
Noor	7(0.3)	4(0.5)	0.378	0.692	75.00	0.475	1.000	100
Mahmoudabad	0(0)	0(0)	-	-	-	-	-	-
Amol	326(16.2)	66(7.6)	0.132	0.679	69.70	0.161	0.903	92.42
Fereydunkenar	23(1.1)	22(2.5)	0.585	0.612	63.64	0.835	0.882	90.91
Babolsar	19(0.9)	18(2.1)	0.288	0.303	33.33	0.576	0.617	77.78
Qaemshahr	155(7.7)	140(16.2)	0.490	0.545	56.43	0.730	0.831	87.14
Sari	648(32.2)	3(0.3)	0.001	0.250	33.33	0.001	0.143	33.33
Neka	30(1.5)	22(2.5)	0.270	0.360	40.91	0.624	0.935	95.45
Behshahr	122(6.1)	107(12.4)	0.502	0.576	59.81	0.822	0.973	98.13
Babol	250(12.4)	250(28.9)	0.690	0.690	70.00	0.942	0.942	95.42
Total Kappa	-	-	0.266	0.632	-	0.347	0.916	-
Total agreement	-	-	27.70%	-	64.51%	39.85%	-	93.45%

* Cancer groups include:1- Lips, mouth and throat 2- Digestive organs 3- Respiratory system and organs inside the chest 4- Bones, joints and articular cartilage 5- Blood systems and hematopoietic organs 6- Skin 7- Soft and connective tissue 8- Breast 9- Female genital organ 10- Male genital organ 11- Urinary system 12- Eyes, brain and other parts of central nervous system 13- Thyroid and other endocrine glands 14- Lymph nodes 15- Unknown primary sites 16- ill-defined sites

**Table 2:** Assessing the accuracy of topographic codes given by pathology centers based on the given codes

**In reports with topographic codes**			**Total reports received**			**Topographic category**	**ICD-O-3**
**Internal reliability N (%)**	**Coding by experts in pathology centers N (%)**	**Coding by experts in cancer registry center N (%)**	**Internal reliability N (%)**	**Coding by experts in pathology centers N (%)**	**Coding by experts in cancer registry center N (%)**		
10 (100)	12 ( 2.0)	10 (1.6)	12 (37.5)	14 (0.4)	32 (1.6)	Lips, oral cavity and throat	C00- C14
246 (97.6)	253 (41.1)	252 (41.0)	345 (51.0)	355 ( 17.6)	679 (33.7)	Digestive organs	C15- C26
4 (100)	4 (0.7)	4 (0.7)	9 (21.4)	9 (0.4)	42 (2.1)	Respiratory system and the chest organs	C30-C39
6 (75.0)	6 (1.0)	8 (1.3)	7 (43.8)	7 (0.3)	16 (0.8)	Bones, joints and articular cartilage	C40- C41
2 (100)	2( 0.3)	2 (0.3)	7 (7.9)	7 (0.3)	89 (4.4)	Blood systems and hematopoietic organs	C42
54 (98.2)	57 (9.3)	55 (8.9)	76 (41.5)	81 (3.9)	183 ( 9.1)	Skin	C44
3 (100)	3 (0.5)	3 (0.5)	3 (30.0)	3 (0.1)	10 (0.5)	Soft and connective tissue	C49
84 (88.4)	88 (14.3)	95 (15.4)	112 (45.3)	118 (5.8)	248 (12.3)	Breast	C50
72 (94.7)	79 (12.8)	76 (12.4)	83 (56.8)	94 (4.6)	147 (7.3)	Female genital organ	C51-C58
38 (84.4)	39 (6.3)	45 (7.3)	54 (39.1)	58 (2.8)	139 (6.9)	Male genital organ	C60-C63
32 (97.0)	36 (5.9)	33 (5.4)	48 (25.5)	53 (2.6)	190 (9.4)	Urinary system	C64-C68
2 (100)	2 (0.3)	2 (0.3)	2 (5.3)	2 (0.1)	38 (1.9)	Eyes, brain and other parts of the central nervous system	C69-C72
2 (100)	2 (0.3)	2 (0.3)	8 (27.6)	8( 0.4)	29 (1.4)	Thyroid and other endocrine glands	C73-C75
2 (100)	4 (0.7)	2 (0.3)	2 (50.0)	5 (0.2)	4 (0.2)	- ill-defined sites	C76
5 (100)	10 (1.6)	5 (0.8)	9 (16.7)	15 (0.7)	55 (2.7)	Lymph nodes	C77
8 (38.1)	18 (2.9)	21 (3.4)	22 (19.5)	36 (1.8)	114 (5.7)	Unknown primary sites	C80
0.906			0.346			Kappa coefficient	
92.68%			39.85			Percentage of total agreement	

**Figure 1:**
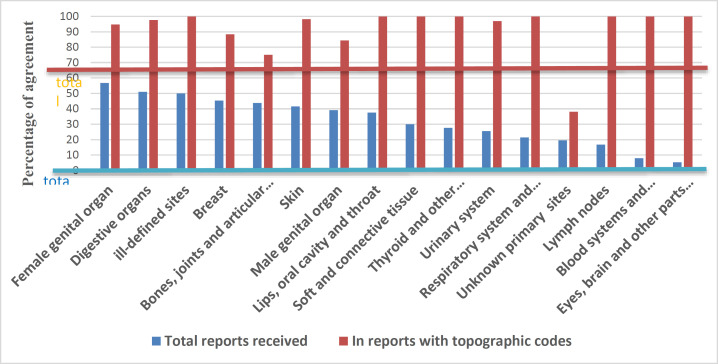
Assessing the percentage of agreement of topographic codes by cancer grouping

**Table 3:** Accuracy of morphology code, behavior and grade of pathology reports by city

**Grade code** ^***^			**Behavior code** ^**^			**Morphology code** ^*^			**City**
**Percentage agreement in reported codes** **(n = 659)**	**Kappa coefficient code in reported codes** **(n = 659)**	**Kappa coefficient in total data** **(n = 2015)**	**Percentage agreement in reported codes** **(n = 865)**	**Kappa coefficient in reported code** **(n = 865)**	**Kappa coefficient in total data** **(n = 2015)**	**Percentage agreement coefficient in reported codes** **(n = 865)**	**Kappa coefficient in reported codes ** **(n = 865)**	**Kappa coefficient** **(n = 2015)** ^****^	
66.15	0.370	0.356	92.31	0.00	0.00	95.38	0.928	0.906	Ramsar
92.86	0.632	0.023	99.24	0.664	0.028	92.37	0.913	0.532	Tonekabon
69.23	0.366	0.025	95.65	0.000	0.014	78.26	0.744	0.141	Chalous
100	1.000	0.082	0.00	0.000	0.000	100	1.000	0.329	Nouhshar
0.000	0.000	0.097	0.00	0.000	0.000	100	1.000	0.512	Nour
-	-	-	-	-	-	-	-	-	Mahmoud Abad
0.000	0.000	0.000	95.45	0.010	0.001	86.36	0.841	0.155	Amol
76.47	0.564	0.339	90.91	0.023	0.030	90.91	0.895	0.851	Fereydunkenar
77.78	0.544	0.481	0.000	0.000	0.000	83.33	0.792	0.744	Babolsar
77.86	0.492	0.395	90.00	0.052	0.043	80.71	0.779	0.695	Qaeimshahr
0.000	0.000	0.000	0.000	0.000	0.000	66.67	0.400	0.002	Sari
54.55	0.272	0.172	95.45	0.000	0.000	95.45	0.936	0.626	Neka
74.29	0.484	0.366	0.000	0.000	0.000	97.20	0.958	0.798	Behshahr
85.94	0.643	0.643	96.80	0.320	0.320	77.20	0.738	0.738	Babol
-	0.531	0.110	-	0.174	0.027	-	0.834	0.331	Total
%79.06	-	%27.34	%96.99	-	%42.98	%86.01	-	%36.92	Total agreement

^*^Morphological codes: carcinoma, lymphoma, leukemia, other specified neoplasms, unspecified neoplasms; ** Behavior codes: benign, uncertain, in situ, malignant; *** Grade codes: grade 0 (no grade), grade 1, grade 2, grade 3, grade 9 (unspecified); **** In total data.

**Table 4:** Accuracy of topographic code, morphology, behavior and grade by type of center (public and private)

**Percentage agreement in reported codes (n=865)**	**Kappa coefficient in reported codes (n=865)**	**Kappa coefficient in total data (n=2015)**	**Type of center**	**Assessment of pathology reports based on**
65.93	0.647	0.283	Governmental	Topographic codes based on accurate code registration
62.60	0.611	0.244	Private	
% 64.51	0.632	0.266	Total	
93.70	0.920	0.367	Governmental	Topographic code based on the grouping of cancers
93.11	0.911	0.321	Private	
% 93.45	0.916	0.347	Total	
84.48	0.818	0.341	Governmental	Morphology code
88.08	0.856	0.319	Private	
% 86.01	0.834	0.331	Total	
96.57	0.176	0.032	Governmental	Behavior Code
97.56	0.170	0.020	Private	
% 96.99	0.174	0.027	Total	
80.90	0.568	0.116	Governmental	Grade Code
76.60	0.484	0.102	Private	
% 79.06	0.531	0.110	Total

## Discussion

The results of the study showed that in the reports received from the pathology centers, based on the accurate topographic codes among all the reports, the accuracy rate was lower than average and among the cities; Babol had the highest accuracy of topographic codes. The total kappa coefficient was acceptable, and the total agreement was above average. The kappa coefficient was relatively good and almost complete in all received reports. In reports with topographic codes, the highest percentage reports of cancers were related to gastrointestinal cancers, breast cancer, female genital cancer and skin cancer. In morphological codes, the accuracy of the codes on the total received reports and the coded reports was relatively good and almost complete, but the accuracy of the behavior and grade codes was very low, even on the reported codes. In general, the reports sent from the pathology centers and also in the reports with the reported codes from the pathology centers, the government centers had a higher accuracy rate and a better performance in registration.

The results of the study showed that based on the registration of the accurate topographic codes among the 2015 reports, the kappa coefficient and the total agreement were 0.266 and 27.70%, which indicates that the accuracy is less than average. Among the cities, Babol with 0.690 (acceptable accuracy), had the highest kappa coefficient. The results of Beam's research aimed at investigating the specificity of international classification codes of diseases in pulmonary bronchial dysplasia using data in electronic health records and a large insurance database showed that the accuracy of each code was from 82 to 95% (16). The results of Khosravi's study showed that 24% of the causes of death registered in hospitals have a classification error and are in the group of absurd causes of death. This has led to the inefficiency of this information in planning and policymaking(17). Also, the Fall's study entitled Death Certificate in Prostate Cancer showed that the proportion of coded prostate cancer reports due to death from prostate cancer in official death certificates was 3% higher than the records reviewed in the prostate cancer registration program(18). 

The present study shows that many reports in Mazandaran University of Medical Sciences are not coded in pathology centers. On the other hand, in Babol University of Medical Sciences, almost all reports are coded in pathology centers, and also if coding is done in pathology centers of these two universities, the accuracy of the codes is generally acceptable. This indicates the accuracy of the coders' coding and regular monitoring of the coding process by the responsible pathologist and the cancer registration team of these universities, especially in Babol University of Medical Sciences. Also, in the pathology centers of Babol University of Medical Sciences, the latest edition of the ICDO3 coding book is available to all pathologists and experts, and regular coding training and retraining courses have been conducted by cancer registration experts of the University of Medical Sciences to cancer registrars in pathology centers. On the other hand, due to the existence of a comprehensive cancer center and the high volume of referrals to pathology centers in Sari city and the lack of staff time in its pathology centers, coding is done in a very small number and with less accuracy in the centers. Also, in the pathology centers of Babol University of Medical Sciences, a large part of the reports are coded under the supervision of the center pathologist, while in the pathology centers of Mazandaran University of Medical Sciences, a large part of the reports by the center coders coded with the help of existing software and without paying attention to ICDO or without supervision of a pathologist.

According to the standardized classification of cancers, in the reports with topographic codes, the most reports of cancers (percentage of agreement) are related to gastrointestinal cancers 246(97.6), breast cancers 84(88.4), female genital cancers 72(94.7) and skin cancer. In Fink's study that was done to assess the accuracy of cancer mortality statistics based on death certificates of 265863 deaths in which cancer was recorded as the main cause of death and the rate of the overall confirmation was 82.8%. This rate was various in coding the main sites of the disease that led to the death. So that the approval rate in some sites was less than 50% and in some sites were 95% or more (19). 

In Johnson's study, assessing the confidence of Qualitative Indicators used in Clinical Cancer Registry Data, concluded that all hospitals currently treating breast cancer patients have the technical capacity to provide data and pathology and surgery data are in high quality. The international classification of diseases, according to the growth of science and the needs of the health community, has always evolved and has played an important role in organizing information and improving treatment processes (20). In Lio's study, assessing the accuracy of disease coding in patients with sarcoma, concluded that the ambiguous definitions of disease could lead to inaccurate coding. As a result, national data sets may not be as comprehensive or useful as expected for studying population-based outcomes for sarcomas (21). The present study confirmed the accuracy of the codes assigned to the reports sent to the cancer registration centers of the northern universities of the country, the reason was the access to consulting pathologists and oncologists in the cancer registration program of these two universities and holding regular meetings to resolve ambiguities in identifying diagnoses and assigning the correct codes to reports. Also, according to the online registration of reports in the cancer registration system of Mazandaran University of Medical Sciences and daily monitoring of the registration of these reports by the cancer registration experts of this university, many problems in registration are fixed immediately and accurately, which increases accuracy and precision. The coding done by the university's cancer registration experts plays an important role. On the other hand, the lack of complexity in diagnosing the affected site in some recurrent cancers can lead to low errors in their topographic coding.

The results showed that the morphology code accuracy on all received reports and coded reports in pathology centers with kappa coefficient of 0.331 and 0.834 that showed a relatively good and almost complete accuracy but the accuracy of the behavior and grade codes, even on reported codes, was very low. The Wener's study was done to assess the data quality indicators in the cancer registry showed that malignant cancers (excluding non-melanoma skin cancers) diagnosed in the country between 1980 and 2014, and the results showed that the incidence of all tumors also increased with certain fluctuations over time (22). The results of Turner's study showed that the research team stated that death certificates accurately identify the cause of death in men with prostate cancer and that their use in the cancer registry program is trustworthy(23). 

In Rashidian's study entitled In-depth exploration of electronic health records stated that these models have the potential to provide coding critiques based on the International Classification of Diseases system that can be used to improve coding accuracy (24). To improve the accuracy of coding the disease, in general, the quality of cancer registration data was acceptable according to the methods presented in this study. The present study also confirmed the accuracy of morphological codes assigned to the reports sent to the cancer registration centers of these medical universities. 

The reason for the inaccuracy of the codes of behavior and grade was their non-registration in pathology centers. Overall, the results of this study show that complete coding of diagnoses has become so important that all hospitals were required to register complete codes of diagnoses in the system (electronic health record), so the correct coding of medical records is essential. Also, the result of the quality of the coded information should be evaluated, and essential steps should be taken to ensure the quality of the coding. Existence of coding errors leads to incorrect classification of diseases and as a result it can cause incorrect statistics. Trust in data whose classification quality is poor, both for care providers and for managers, is a risk in planning and policymaking, educational and research affairs, care and reimbursement.

The results of the accuracy of the topographic, morphology, behavior and grade codes by center types (public and private), showed that the accuracy is less than average and in all cases, governmental centers have a higher accuracy in registration. The study of Jahanbakhsh stated that in all educational and non-educational centers, the average knowledge of coders is less than 50% and indicates a low level of knowledge of coders of the rules and principles of coding (25). The study of Abzari stated that the staff of public and private hospitals had different motivational priorities. Therefore, the managers of these organizations should understand these differences in different employees and try to motivate them (26). 

The study of Beasley stated that the staff of health information management in hospital does not only work in this department, but also works in other departments, that each department requires its own specialized skills(27). In general, it can be said that one of the reasons for the inaccuracy of behavior and grade codes is the lack of coded reports in centers, especially the lack of coded reports in private centers. Also, many coders in pathology centers do not have the relevant academic education to perform accurate coding. Therefore, special attention should be paid to holding training courses as well as creating motivational factors for employees in private pathology centers to register these codes more and more accurately and improve the quality of coding. Also, if special attention is paid to the inclusion of code of behavior and grade in the integrated hospital system, this can improve the more accurate recording of these codes in pathology reports and hospital records of patients.

One of the limitations of this research is the incompleteness of collecting all pathology reports in these three years from pathology centers, and also the lack of coding of all pathology reports in pathology centers. The accuracy of the codes given in the pathology centers by cities is in their measurement with the codes given by the coder of the Cancer Registration Center is moderate in topographic codes, is moderate in morphological codes, is low in behavior codes and is moderate in grade codes. According to the results of the study, if the coding of reports in pathology centers is complete, the available data can be considered reliable for use in epidemiological studies and other research related to cancer. It is recommended to carry out educational interventions to empower and increase the skills of coders, to motivate them to perform more complete coding in pathology centers, as a result of increasing the coding quality of pathology reports. If the coding is done completely and correctly in the pathology centers, they do not need to be re-coded in the cancer registration centers of medical universities, and instead the cancer registration experts can spend more time to monitor and train the coders of the pathology centers.
